# Ligand‐targeted polymerase chain reaction for the detection of folate receptor‐positive circulating tumour cells as a potential diagnostic biomarker for pancreatic cancer

**DOI:** 10.1111/cpr.12880

**Published:** 2020-07-24

**Authors:** Hao Cheng, Wei He, Jun Yang, Qing Ye, Lu Cheng, Yiming Pan, Liang Mao, Xuehui Chu, Chenglin Lu, Gang Li, Yudong Qiu, Jian He

**Affiliations:** ^1^ Nanjing Drum Tower Hospital Clinical College of Nanjing Medical University Nanjing China; ^2^ Department of Hepatobiliary and Pancreatic Surgery Nanjing Drum Tower Hospital The Affiliated Hospital of Nanjing University Medical School Nanjing China; ^3^ Department of Medicine Geno Biotech Co Ltd Shanghai China; ^4^ Department of Pathology Nanjing Drum Tower Hospital The Affiliated Hospital of Nanjing University Medical School Nanjing China; ^5^ Department of Research and Development Geno Biotech Co Ltd Shanghai China; ^6^ Department of Radiology Nanjing Drum Tower Hospital The Affiliated Hospital of Nanjing University Medical School Nanjing China

**Keywords:** circulating tumour cells, folate receptor, ligand‐targeted polymerase chain reaction, pancreatic cancer, periampullary cancer

## Abstract

**Objectives:**

To detect folate receptor (FR)‐positive circulating tumour cells (FR^+^ CTCs) by using ligand‐targeted polymerase chain reaction (LT‐PCR) in periampullary cancer patients and to investigate the diagnostic value of FR^+^ CTCs in distinguishing pancreatic cancer (PC) from benign pancreatic disease.

**Materials and Methods:**

CTCs were enriched from 3 mL of peripheral blood and portal vein blood by immunomagnetic depletion of leucocytes and were then detected by LT‐PCR. The diagnostic performance of FR^+^ CTCs in PC was investigated by receiver‐operating characteristic curve analysis.

**Results:**

In total, 57 consecutive patients, including 46 patients with PC, five patients with non‐pancreatic periampullary cancer (non‐PC) and six patients with benign pancreatic diseases, were enrolled. FR^+^ CTC levels were significantly higher in patients with malignant diseases (PC and non‐PC) than in patients with benign pancreatic diseases (*P* < .01). There was no notable difference in CTC levels between patients with PC and those with non‐PC (*P* > .05). The combination of FR^+^ CTCs with carbohydrate antigen 19‐9 (CA19‐9) had better diagnostic efficiency than each of these two markers alone, with high sensitivity (97.8%) and specificity (83.3%).

**Conclusions:**

LT‐PCR is feasible and reliable for detecting FR^+^ CTCs in patients with periampullary cancer. FR^+^ CTCs, especially when used in combination with CA19‐9, have potential as a biomarker for the diagnosis of PC.

## Graphical abstract

Ligand‐targeted PCR is feasible and reliable for detecting FR^+^ CTCs. FR^+^ CTCs, especially when used in combination with CA19‐9, have potential as a biomarker for the diagnosis of pancreatic cancer.

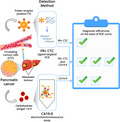

## 1 INTRODUCTION

Pancreatic, distal bile duct, ampullary and duodenal cancers are the main types of periampullary cancers.^1^ They constitute an entity of aggressive tumours and the 5‐year survival rate after operation with curative intent has improved very little over the last two decades.^2^ Among them, pancreatic cancer (PC) is historically characterized by increasing mortality rates and is currently predicted to become the second leading cause of cancer‐related deaths in the United States by 2030.^3^, ^4^ Due to late presentation and early metastasis, PC is largely diagnosed at an advanced stage, and therefore, the prognosis generally is poor.^5^ Early diagnosis is mostly hampered by the lack of serum biomarkers with high diagnostic sensitivity and specificity.^6^ Carbohydrate antigen 19‐9 (CA19‐9) currently is the only biomarker approved by the United States Food and Drug Administration (FDA) and is widely used in clinical practice; however, it is insufficiently sensitive for early diagnostic screening and non‐specific, as CA19‐9 levels are elevated in a number of benign pancreatic and biliary diseases.^7^ Thus, reliable biomarkers capable of efficiently establishing diagnosis at the time of disease presentation remain an urgent need.

Circulating tumour cells (CTCs) were postulated by Paget to act as essential “seeds” of metastases in breast cancer more than a century ago.^8^ The “seed and soil” hypothesis suggests that CTCs, that is tumour cells that escaped from primary tumours into the circulation through invading adjacent vasculature, disseminate to colonize distant sites.^9^, ^10^ Accumulating evidence has demonstrated that CTCs have wide clinical application prospects, including early cancer detection, disease monitoring, prognosis and identification of therapeutic targets.^11^, ^12^, ^13^ Recent advances in detection technologies and the characterization of CTCs have improved our understanding of the biology of CTCs and their role in cancer dissemination.^14^, ^15^ As a liquid biopsy, CTC counting has been included in the National Comprehensive Cancer Network guidelines and is defined as a prognostic factor in breast cancer by the American Joint Committee on Cancer Staging. Especially in tumours that are difficult to access, such as periampullary cancers, the potential value of CTC detection has been established and become increasingly important.^1^

The CellSearch System is the first and currently only FDA‐approved CTC detection platform. It uses an immunomagnetic method for quantitative evaluation of CTCs by capturing the epithelial cell marker, epithelial cell adhesion molecule (EpCAM).^16^ Among different types of metastatic cancers, pancreatic ductal adenocarcinoma (PDAC) exhibited the lowest detection rate and CTC counts in a study evaluating the efficiency of the CellSearch System in nearly 1000 patients, and this was in line with findings in other studies.^17^, ^18^, ^19^ Another important limitation of this system is that it cannot detect CTCs with non‐epithelial characteristics (EpCAM‐negative CTCs). The non‐immunologic‐based and EpCAM‐independent CTC isolation technique termed “isolation by size of epithelial tumour cells” (“ISET”) has a better detection efficiency than the CellSearch System in PC^20^; however, it may miss smaller cells and requires validation before clinical application.

Folate receptors (FRs) are cysteine‐rich cell‐surface glycoproteins that bind folate with high affinity to mediate cellular uptake of folate.^21^ To meet the folate demand of rapidly dividing cells under low‐folate conditions, FRs are highly expressed in a variety of cancers, including PC, whereas most normal tissues express these receptors at low to negligible levels.^22^, ^23^, ^24^ In the circulation, there are no normal cells, except a subgroup of activated macrophages, that express functional FRs.^25^ However, activated macrophages are barely detectable in blood samples from healthy donors or from patients with benign diseases.^26^ Therefore, FRs may be a potential target for capturing CTCs in patients with PC.

Ligand‐targeted polymerase chain reaction (LT‐PCR), developed by Geno Biotech, has shown promise in detecting CTCs in non‐small cell lung cancer through targeting FRs on the cell surface. In this method, CTCs are labelled with a conjugate of a tumour‐specific ligand (folic acid) selectively bound to cancer cells over‐expressing FRs and a synthetic oligonucleotide. The conjugate serves as an adapter molecule to convert a CTC into detector “oligonucleotides” that can be amplified for quantitative analysis. LT‐PCR allowed specific detection of CTCs in healthy blood spiked with tumour cells expressing FRs on their surface, and a clinical study showed that the method was sufficiently sensitive to stratify patients with different disease statuses.^27^ Potential advantages of LT‐PCR over other CTC detection techniques include ultrasensitive analysis that enables advanced disease stratification, low sampling volume (3 mL) and a standardized platform that eliminates artificial analysis bias. In the present study, we applied LT‐PCR to detect FR‐positive (FR^+^) CTCs in periampullary cancer patients and analysed their clinical significance in the diagnosis of PC.

## 2 MATERIALS AND METHODS

### 2.1 Patients and clinical data collection

Between August 2018 and December 2019, 51 consecutive patients with newly confirmed periampullary cancer were enrolled into this prospective study. None of the included patients had received any anticancer therapies or had a history of any other malignancy during the past five years. Diagnosis was based on histopathologic observations of specimens obtained by fine‐needle aspiration (FNA) or resection. Disease stages were based on the eighth edition of the American Joint Committee on Cancer Staging manual. Clinical data, including age, gender, preoperative CA19‐9 and bilirubin levels, location of primary tumour, tumour differentiation, tumour size, lymph‐node metastasis and distant metastases, were collected. In addition, six patients with benign pancreatic diseases were enrolled into the study. The study was approved by the Ethics Committee of Nanjing Drum Tower Hospital, and informed written consent was obtained from all subjects before the study. All procedures were in accordance with the guidelines of the Declaration of Helsinki.

### 2.2 Blood sample processing

Peripheral blood samples (3 mL) from eligible individuals were collected in vacuum tubes containing the anticoagulant ethylenediaminetetraacetic acid for analysis before commencing treatment. In a subset of patients, paired portal venous blood was drawn intraoperatively or by endoscopic ultrasound‐guided (EUS‐)FNA. All blood specimens were stored at 4°C and processed within 24 hours of blood withdrawal.

### 2.3 CTC detection

**FIGURE 1:**
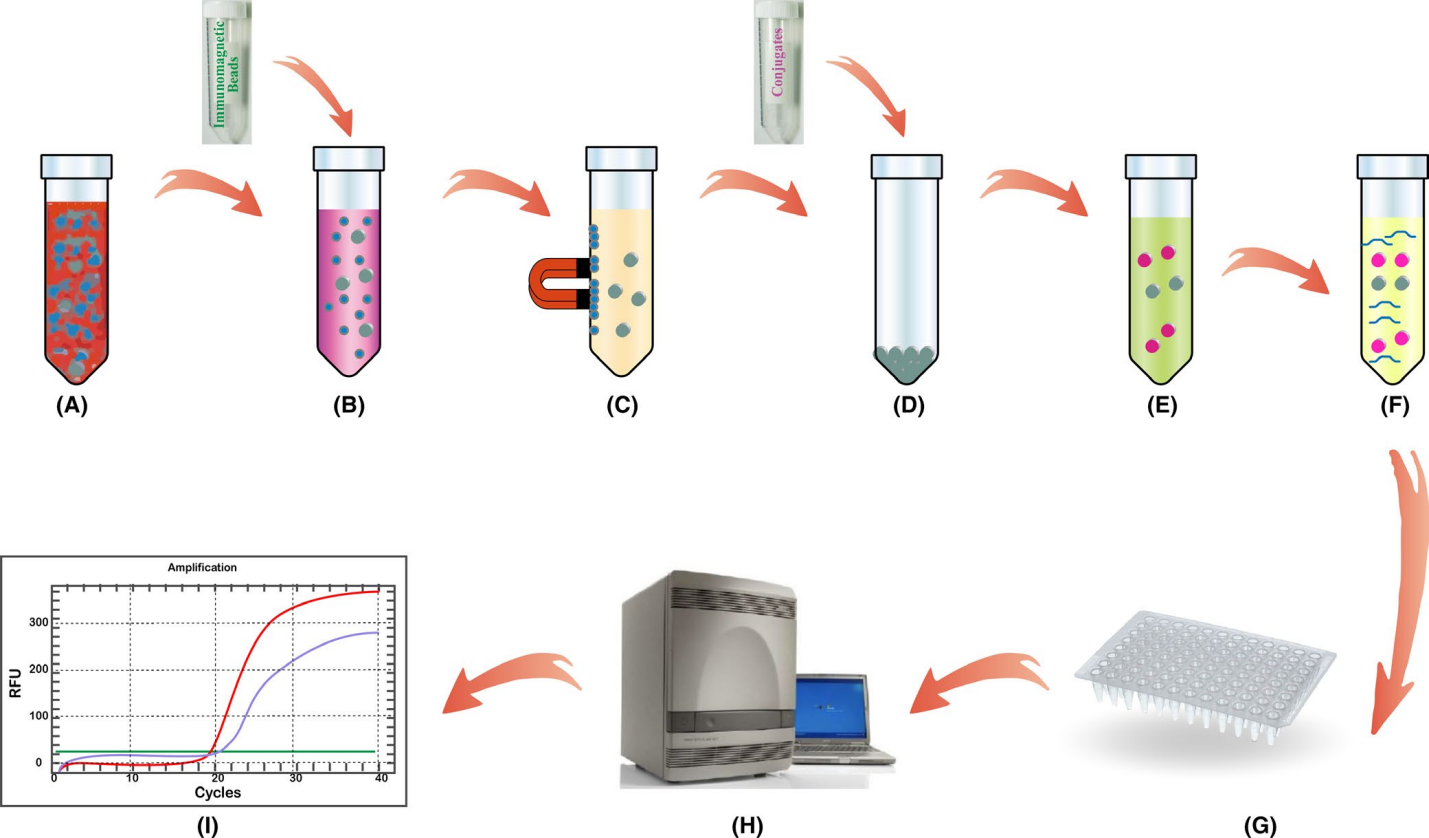
Flow chart of the CytoploRare protocol: A, lysis of erythrocytes; B, incubation with immunomagnetic beads (anti‐CD45 and anti‐CD14); C, immunomagnetic depletion of leucocytes; D, incubation with conjugates of a tumour‐specific ligand, folic acid and a synthetic oligonucleotide; E, removal of unbound conjugates; F, eluting of the probe from CTCs; G, transfer of the samples into the PCR system; H, PCR amplification; I, data analysis

CTCs were enriched and enumerated using the CytoploRare Kit (Geno Biotech), a commercially available CTC detection kit approved by the China FDA, as described previously.^26^, ^28^ The method comprises two major steps: CTC enrichment based on negative enrichment by immunomagnetic beads, and CTC detection and quantification based on LT‐PCR. The experimental procedure was performed strictly according to the manufacturer's protocol. Briefly, the whole blood samples were first incubated with 12 mL of lysing buffer and anti‐CD45/‐CD14 immunomagnetic beads to lyse erythrocytes and remove leucocytes, respectively. The enriched samples were then incubated with 10 μL of detection probes at room temperature for 40 minutes. The detection probe with a FR alpha‐targeting folic acid unit was designed to specifically label FR‐expressing cells. It also comprises an oligonucleotide unit (5′‐CTCAA CTGGT GTCGT GGAGT CGGCA ATTCA GTTGA GGGTT CTAA‐3′) for subsequent PCR amplification. After repeated washing, the detection probes were eluted. Fluorescence quantitative PCRs to amplify and quantify the signals from the detection probes were run using the ABI 7300 Real‐Time PCR System (Thermo Fisher). The thermal cycles were as follows: denaturation at 95°C for 2 minutes, annealing at 40°C for 30 seconds, extension at 60°C for 1 minute, cooling at 8°C for 5 minutes, followed by denaturation at 95°C for 1 minute and 40 cycles of denaturation at 95°C for 10 seconds, annealing at 35°C for 30 seconds and extension at 72°C for 5 seconds. Primer and probe sequences were as follows: forward primer 5′‐TATGA TTATG AGGCA TGA‐3′; reverse primer 5′‐GGTGT CGTGG AGTCG‐3′; TaqMan probe 5′‐FAM‐CAGTT GAGGG TTC‐MGB‐3′. The level of FR^+^ CTCs in each sample was calculated on the basis of a calibration curve generated with the standard reference materials provided in the kit (serially diluted oligonucleotides corresponding to 2.00‐632.50 folate units). FR^+^ CTC levels were measured in folate units (FU)/3 mL of blood (Figure 1).

### 2.4 Measurement of CA19‐9

CA19‐9 expression levels in all blood samples were determined by electrochemiluminescence assay (Roche). The reference value range for the biomarker was 0‐27 U/mL.

### 2.5 Statistical analysis

Data were analysed using IBM SPSS, v.25 (IBM Corp.), and graphs were prepared using PRISM 8 (GraphPad Software, Inc). Continuous variables were summarized as means or medians and ranges, respectively. To compare CTC levels between two groups, we used the Mann‐Whitney *U* test, and to compare the levels between three groups, the Kruskal‐Wallis test was used. Receiver‐operating characteristic (ROC) curves were used to determine thresholds associated with high sensitivity and specificity, and the area under the ROC curve (AUROC) was calculated for each index. The Youden index was used to identify the optimal cut‐off point and diagnostic efficiency. All *P* values were based on two‐sided testing, and *P* < .05 was considered significant.

## 3 RESULTS

### 3.1 Patient characteristics

Patient characteristics are summarized in Table 1. The patients with PC included in this study had a mean age of 65 years (range, 42‐87 years) and were predominantly males (n = 27, 60%). More than 50% of the tumours were located in the head or neck, and according to the histopathologic type, most were PDAC, and only two were malignant intraductal pancreatic mucinous neoplasia (IPMN). There were five patients with non‐pancreatic periampullary cancer (non‐PC), including three with ampullary cancer (AMPC) and two with distal bile duct cancer (DBDC). In the benign diseases group, 50% were pancreatic cystic neoplasm. The clinical data of one patient with PC were missing.

**TABLE 1:** Patient characteristics

Characteristics	No. of patients
PC (n = 45)	
Age, years mean (range)	65 (42‐87)
Sex, male	27 (60.0%)
Tumour size, mm mean (range)	36 (12‐95)
Tumour location	
Head or neck	24 (53.3%)
Body or tail	21 (46.7%)
Tumour stage	
I	10 (22.2%)
II	14 (31.1%)
III	13 (28.9%)
IV	8 (17.8%)
Tumour differentiation	
Well	16 (35.6%)
Moderate	4 (8.9%)
Poor	25 (55.5%)
Vascular infiltration	
Yes	26 (57.8%)
No	19 (42.2%)
Histopathologic type	
PDAC	43 (95.6%)
Malignant IPMN	2 (4.4%)
Non‐PC (n = 5)	
Age, years mean (range)	59 (46‐71)
Sex, male	4 (80.0%)
Tumour size, mm mean (range)	22 (12‐42)
Tumour stage	
I	0
II	2 (40.0%)
III	2 (40.0%)
IV	1 (20.0%)
Tumour differentiation	
Well	1 (20.0%)
Moderate	3 (60.0%)
Poor	1 (20.0%)
Histopathologic type	
AMPC	3 (60%)
DBDC	2 (40%)
Benign diseases (n = 6)	
Age, years mean (range)	63 (53‐74)
Sex, male	4 (66.7%)
Histopathologic type	
Cyst	2 (33.2%)
IPMN	1 (16.7%)
MCN	1 (16.7%)
SCN	1 (16.7%)
CP	1 (16.7%)

Abbreviations: AMPC, ampullary cancer; CP, chronic pancreatitis; DBDC, distal bile duct cancer; IPMN, intraductal pancreatic mucinous neoplasia; MCN, mucinous cystic neoplasm; Non‐PC, non‐pancreatic periampullary cancer; PC, pancreatic cancer; PDAC, pancreatic ductal adenocarcinoma; SCN, serous cystic neoplasm.

### 3.2 FR^+^ CTC levels in patients with periampullary cancer and benign diseases

**FIGURE 2:**
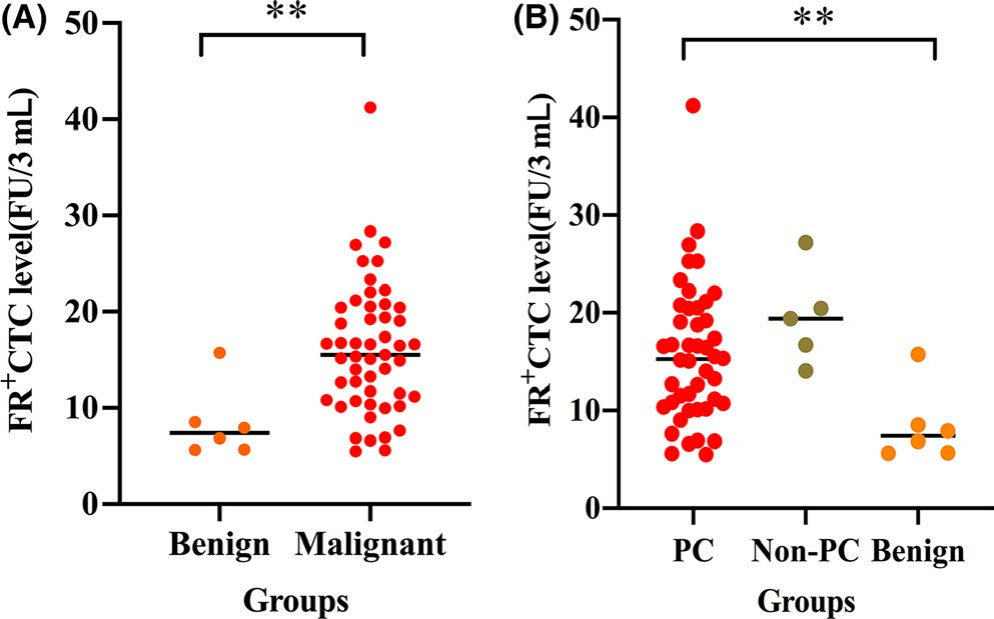
FR^+^ CTC levels in patients with periampullary cancers and benign diseases. A, Comparison of CTC levels in patients with benign and malignant diseases. B, Comparison of CTC levels in patients with PC, non‐PC and benign diseases. FR^+^ CTC, folate receptor‐positive circulating tumour cell; PC, pancreatic cancer; non‐PC, non‐pancreatic periampullary cancer. **P < .01

CTC levels are presented as medians and interquartile ranges (IQRs). They were 15.26 FU/3 mL (10.78‐20.13 FU/3 mL), 19.40 FU/3 mL (16.74‐20.44 FU/3 mL) and 7.38 FU/3 mL (5.96‐8.36 FU/3 mL) in patients with PC, non‐PC and benign pancreatic diseases, respectively. First, we compared the CTC levels between the malignant (PC and non‐PC) and benign groups. The CTC levels in patients with malignant diseases (15.53 FU/3 mL, 11.01‐20.44 FU/3 mL) were significantly higher than those in patients with benign pancreatic diseases (*P* < .01; Figure 2A). Next, the CTC levels were compared among all three groups. A statistically significant difference between the three groups was found (*P* < .01; Figure 2B). The CTC levels in patients with benign pancreatic diseases were significantly lower than those in patients with PC (*P* < .01) and non‐PC (*P* < .01); however, there was no notable difference between the levels in patients with PC and those in patients with non‐PC (*P* > .05; Table 2).

**TABLE 2:** Comparison of CTC levels in the different groups (PC, non‐PC, and benign diseases)

Groups	Mean difference	SE	*P*
PC vs non‐PC	–11.07	7.22	>.05
PC vs benign diseases	19.03	6.66	<.01
Non‐PC vs benign diseases	30.10	9.29	<.01

Abbreviations: CTC, circulating tumour cell; non‐PC, non‐pancreatic periampullary cancer; PC, pancreatic cancer; SE, standard error.

We also analysed the correlation between CTC levels and clinical characteristics of patients with PC, and the results are shown in Table 3. We found no correlation between the CTC levels and clinical characteristics, including preoperative bilirubin, tumour location, tumour size, tumour stage, lymph‐node status and vascular infiltration.

**TABLE 3:** Relationships between CTC levels and clinicopathological characteristics of patients with PC

Characteristic	No. of patients	CTC units, median (interquartile range)	*P*
Sex			
Female	18	15.13 (9.83‐16.66)	>.05
Male	27	16.45 (10.73‐22.04)	
Age (y)			
≤60	12	15.97 (10.17‐22.65)	>.05
>60	33	15.08 (10.61‐19.13)	
Preoperative CA19‐9 level U/mL			
≤27	11	16.47 (13.26‐18.75)	>.05
>27	34	15.05 (10.13‐20.46)	
Preoperative bilirubin level μmol/L			
≤28	33	14.01 (10.24‐18.91)	>.05
>28	12	16.55 (7.85‐21.08)	
WBC (×10^9^/L)			
≤6.5	33	15.53 (11.61‐19.83)	>.05
>6.5	12	10.61 (9.25‐20.05)	
Tumour location			
Head or neck	24	15.44 (11.30‐18.41)	>.05
Body or tail	21	13.26 (10.14‐21.38)	
Tumour size (mm)			
≤40	28	15.44 (10.78‐20.10)	>.05
>40	17	12.70 (6.36‐20.19)	
Tumour stage			
I	10	15.22 (13.82‐20.53)	>.05
II	14	12.68 (9.21‐18.87)	
III	13	16.61 (10.14‐23.76)	
IV	8	15.97 (9.35‐19.74)	
Tumour differentiation			
Well	16	14.11 (7.00‐18.64)	>.05
Moderate	4	12.59 (6.58‐22.46)	
Poor	25	15.35 (10.79‐20.49)	
Lymph‐node status			
N0	13	14.01 (11.00‐17.28)	>.05
N1 + N2	10	16.55 (6.90‐19.90)	
Margins			
R0	13	14.92 (11.75‐20.01)	>.05
R1	10	13.13 (9.30‐17.16)	
Vascular infiltration			
No	19	14.92 (9.01‐16.76)	>.05
Yes	26	16.54 (11.23‐21.39)	
Metastasis			
No	37	15.08 (10.79‐19.82)	>.05
Yes	8	15.97 ( 9.35‐19.74)	

Abbreviations: CA19‐9, carbohydrate antigen 19‐9; CTC, circulating tumour cell; PC, pancreatic cancer; WBC, white blood cell.

### 3.3 FR^+^ CTC levels in peripheral blood and portal vein blood

**FIGURE 3:**
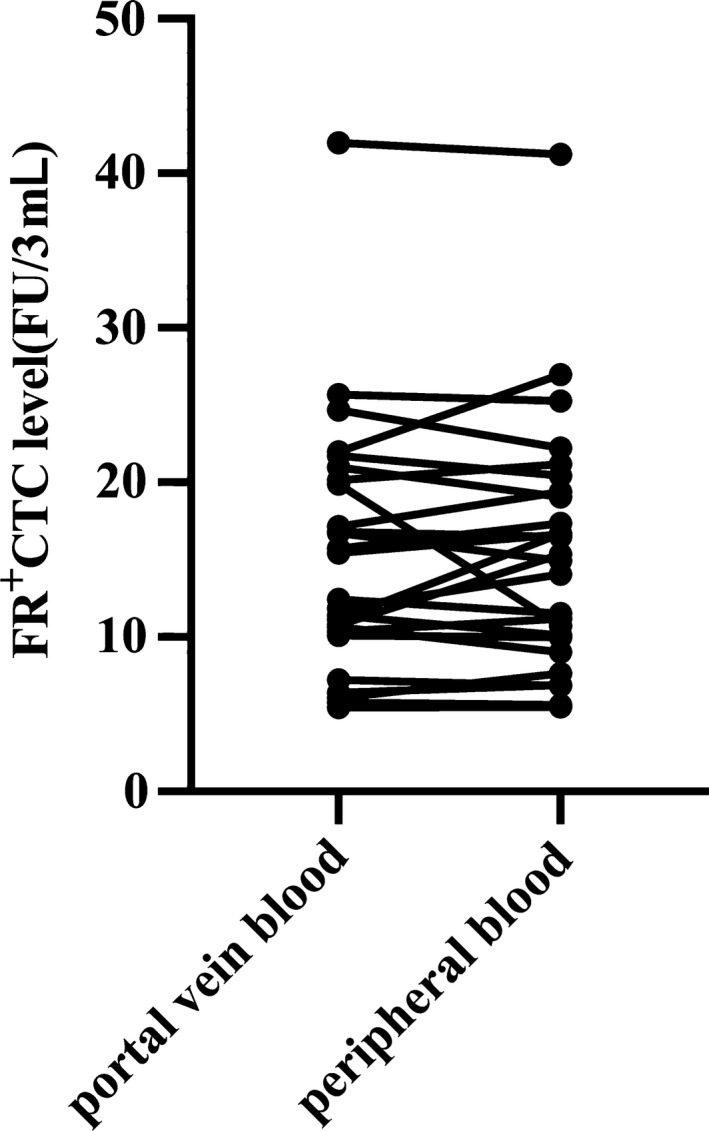
Comparison of FR^+^ CTC levels in portal vein and peripheral blood samples. FR^+^ CTC, folate receptor‐positive circulating tumour cell

Next, we aimed to determine whether CTC levels detected by LT‐PCR are higher in portal vein blood than in peripheral blood, as reported for other detection methods in patients with periampullary cancer or PC.^29^, ^30^ In total, 27 patients, including three patients with non‐PC and 24 patients with PC, had portal venous blood drawn intraoperatively or by EUS‐FNA. Notably, the CTC levels in portal vein blood (15.42 FU/3 mL, 10.50‐20.02 FU/3 mL) were slightly higher than those in peripheral blood (15.35 FU/3 mL, 10.04‐19.23 FU/3 mL), although the difference was not significant (*P* > .05) as indicated by paired analysis (Figure 3).

### 3.4 Diagnostic efficiencies of FR^+^ CTC and CA19‐9 levels and their combination for PC

**FIGURE 4:**
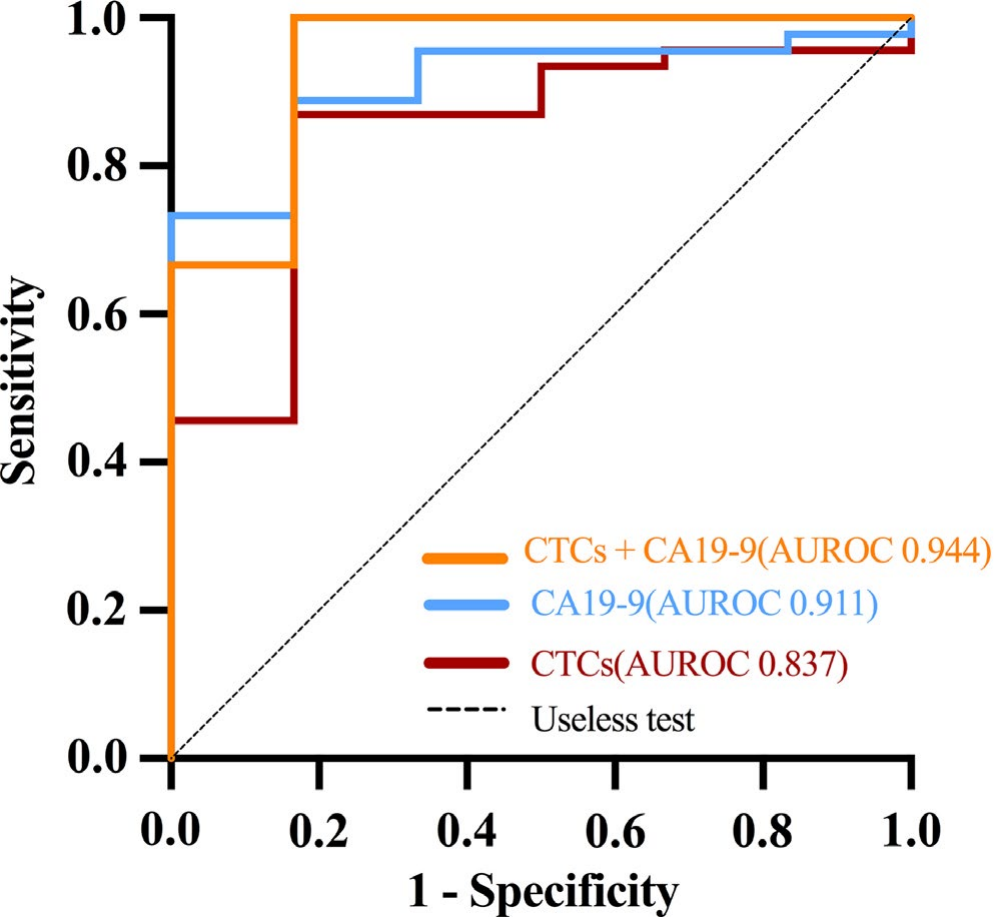
ROC curves showing the diagnostic performance of FR^+^ CTCs, CA19‐9 and their combination. FR^+^ CTCs, folate receptor‐positive circulating tumour cells; CA19‐9, carbohydrate antigen 19‐9; ROC, receiver‐operating characteristic; AUROC, area under the ROC curve

The diagnostic efficiencies of FR^+^ CTC and CA19‐9 levels in distinguishing PC from benign pancreatic diseases were first compared on the basis of ROC curves. The optimal FR^+^ CTC level cut‐off value for differentiating patients with PC from those with benign pancreatic diseases was 8.76 FU/3 mL, with a sensitivity of 86.7% and a specificity of 83.3%, and the AUROC was 0.837 (95% confidence interval [CI], 0.675‐0.999). However, the AUROC of CA19‐9 was 0.911 (95% CI, 0.819‐1.000). Thus, compared with CA19‐9, the diagnostic value of the FR^+^ CTC level was slightly poorer (Table 4). To improve the diagnostic accuracy, we tested the combination of FR^+^ CTC and CA19‐9 levels for PC diagnosis. Notably, using the combination significantly improved the diagnostic efficiency in differentiating patients with PC from those with benign pancreatic diseases when compared to CTCs or CA19‐9 alone (Figure 4).

**TABLE 4:** Diagnostic efficiencies of FR^+^ CTCs, CA19‐9, and their combination in differentiating patients with PC and pancreatic benign diseases

Method	Sensitivity	Specificity	AUROC (95% CI)	*P*
FR^+^ CTCs	0.867	0.833	0.837 (0.675‐0.999)	<.05
CA19‐9	0.733	1.000	0.911 (0.819‐1.000)	<.05
FR^+^ CTCs+CA19‐9	0.978	0.833	0.944 (0.840‐1.000)	<.001

Abbreviations: AUROC, area under the receiver‐operating characteristic curve; CA19‐9, carbohydrate antigen 19‐9; CI, confidence interval; FR^+^ CTCs, folate receptor‐positive circulating tumour cells; PC, pancreatic cancer.

## 4 DISCUSSION

In the present study, we found that FR^+^ CTC levels were significantly higher in patients with periampullary cancers, including PC, than in patients with benign pancreatic diseases. FR^+^ CTC levels could effectively distinguish PC from benign diseases with a high AUROC value, albeit with lower efficiency than CA19‐9; however, the diagnostic efficiency of FR^+^ CTCs for PC improved when combined with CA19‐9.

CTCs have been recently demonstrated to have high potential value in diagnosing, evaluating clinical effects and monitoring recurrence in patients with various malignancies, and as a liquid biopsy, they are of particular interest given their location in the vasculature, allowing for easy sampling and analysis.^31^ In recent years, sensitive and reproducible platforms have been developed for the detection, isolation and enrichment of CTCs from peripheral blood; however, methodological standardization is required for such technologies to have a significant effect on clinical care.^32^ Our previous studies, including large‐scale, prospective, double‐blind clinical trials, have shown the promising clinical value of detecting FR^+^ CTCs by LT‐PCR—based on negative depletion of leucocytes followed by labelling with folate‐linked oligonucleotide and quantification by quantitative PCR—in non‐small cell lung cancer patients.^33^, ^34^, ^35^, ^36^ As FRs are also highly expressed in PC, in the current study, we used the LT‐PCR technique to identify FR^+^ CTCs in periampullary cancers, including PC. To our knowledge, this is the first study to do so. To deplete activated macrophages in blood samples, anti‐CD14‐coated magnetic beads were used after depletion of CD45‐positive leucocytes.

CTC levels in patients with periampullary cancer were significantly higher than those in patients with benign pancreatic diseases, whereas there was no difference between PC and non‐PC patients. In our previous studies, median CTC levels in healthy donors were consistently <6 FU/3 mL and were significantly lower than those in patients with malignant diseases^28^, ^33^, ^37^; therefore, we did not recruit healthy donors in the current study. Two patients with DBDC were included in our study, and both had CTC levels >20 FU/3 mL. Yang and colleagues found that CTCs detected by the CellSearch System were associated with more aggressive tumour characteristics and were independently associated with survival in patients with cholangiocarcinoma; however, one or more CTC was detected in only 28% of patients.^38^ Hence, we hypothesize that the FR^+^ CTCs detected by LT‐PCR might also be a potential biomarker for cholangiocarcinoma, based on our very limited results.

The diameter of CTCs is around 25 µm, which is far too large to allow them to pass through the capillaries (8 µm diameter).^10^ The portal vein drains periampullary and pancreatic tissues, and theoretically, CTCs will be more easily detectable in portal than in peripheral venous blood of patients with periampullary or pancreatic cancer. Several studies have tested this hypothesis. The results consistently showed that when using the CellSearch System or other techniques, CTCs were detected at higher rates and at significantly higher numbers in portal than in peripheral venous blood, and the CTC count in portal venous blood was a significant predictor for early liver metastases after curative resection in patients with periampullary or pancreatic cancer.^29^, ^30^ In addition, it is feasible and safe to collect portal venous blood from patients undergoing EUS‐FNA.^39^ In our study, under EUS guidance, a 19‐gauge EUS fine needle was advanced transhepatically into the portal vein, and in this way, 3 mL of blood was aspirated safely from seven patients. However, we found no significant difference in the CTC level between portal vein and in peripheral blood by paired analysis, which was not in line with the findings in previous studies.^29^, ^30^ One of the reasons may be the different CTC detection methods used. Parker et al detected >100 000 FRs on the surface of one CTC cell by using a quantitative radioligand binding assay.^24^ In our method, CTCs in the blood was enriched by nearly 100 000 orders of magnitude through binding of the folate‐linked oligonucleotide with the FR, and then, the targeted FRs were amplified by RT‐PCR. This two‐step enrichment may have masked differences in CTC counts between portal vein and peripheral blood.

PC is often detected only in advanced stages and has an aggressive biology; therefore, it is associated with a high mortality rate. Most patients have no early symptoms and initially present with mild symptoms, such as tiredness, weight loss, abdominal discomfort and back pain. Thus, there is a need for biomarkers that can help efficiently establish diagnosis at the time of disease presentation. To date, only serum CA19‐9 is routinely used as a non‐invasive blood‐based biomarker in PC. However, elevated CA19‐9 expression is detected in various benign diseases, especially in diseases with obstructive jaundice (acute cholangitis and bile duct stones) and malignant (colorectal cancer, gastric cancer, bladder cancer and uterine squamous cell carcinoma) diseases in addition to PC, and thus, this marker is non‐specific. Additionally, CA19‐9 is a sialylated Lewis‐blood‐group antigen, and approximately 10% of the general population is—by inheritance—negative for this antigen and, thus, will not express CA19‐9 if they develop pancreatic carcinoma.^40^ In this study, we investigated the diagnostic value of FR^+^ CTCs in PC and compared it with that of CA19‐9. Our results showed that CTC levels detected by LT‐PCR displayed a slightly lower AUROC, with a sensitivity of 86.7% and a specificity of 83.3%, than CA19‐9 in the diagnosis of PC. Encouragingly, compared to CTCs or CA19‐9 alone, the diagnostic efficiency improved when these two markers were combined in the diagnosis of PC, with a sensitivity of 97.8% and a specificity of 83.3%. We found no correlation between CTC levels and preoperative bilirubin levels, which overcomes one of the shortcomings of CA19‐9. Thus, FR^+^ CTCs may serve as an effective auxiliary diagnostic marker for PC, and their combination should be recommended in clinical practice in future.

In conclusion, this small‐scale, exploratory clinical trial revealed that LT‐PCR is feasible and reliable for detecting FR^+^ CTCs in patients with periampullary cancers, including PC. This strategy complements the current standard EpCAM‐based CTC detection. More importantly, FR^+^ CTCs may serve as a useful diagnostic biomarker for patients with PC, especially when used in combination with CA19‐9. Further study to investigate the clinical significance of CTCs detected by LT‐PCR in a larger cohort is warranted.

## CONFLICT OF INTEREST

The authors declare no competing interests.

## AUTHOR CONTRIBUTIONS

HC, WH, YQ and JH design the study. LC involved in contribution of CTC detection method and reagents. JY, QY, HC, YP, LM, XC, CL and GL collected the data. HC analysed the data. HC prepared the manuscript. YQ and JH involved in funding. All authors interpreted results and contributed to the manuscript.

## Data Availability

The data that support the findings of this study are available from the corresponding author upon reasonable request.
